# Evaluating the impact of an assertiveness communication training programme for Japanese nursing students: A quasi‐experimental study

**DOI:** 10.1002/nop2.228

**Published:** 2018-12-09

**Authors:** Mieko Omura, Tracy Levett‐Jones, Teresa E. Stone

**Affiliations:** ^1^ School of Nursing and Midwifery, Faculty of Health and Medicine The University of Newcastle Callaghan New South Wales Australia; ^2^ Faculty of Health University of Technology Sydney Ultimo New South Wales Australia; ^3^ Faculty of Nursing Chiang Mai University Chiang Mai Thailand

**Keywords:** assertiveness, attitude, communication, education, Japan, nursing, speak up, student, Theory of Planned Behaviour, training

## Abstract

**Aim:**

To examine the impact of an assertiveness communication training programme on Japanese nursing students’ level of assertiveness and intention to speak up when concerned about patient safety.

**Design:**

A quasi‐experimental design with two parallel groups was used.

**Methods:**

Third‐year nursing students from two Japanese educational institutions were allocated to an intervention and control group. The intervention group completed the Theory of Planned Behaviour–Assertive Communication Questionnaire and the Japanese version of the Rathus Assertiveness Schedule after attending assertive communication workshops. The second group completed the surveys before attending the workshop and were referred as the control group. Data were collected in December 2017–January 2018.

**Results:**

A total of 123 out of 150 nursing students completed the surveys. Following the assertiveness communication training programme, a higher percentage of students from the intervention group demonstrated the intention to speak up. The intervention group also displayed higher levels of assertiveness, although the results were not statistically significant.

## 1 INTRODUCTION

Assertive communication has been recognized as a key strategy for preventing adverse incidents in health care and improving patient safety (Lo, 2011). During the past decade, researchers have focused on improving healthcare professionals’ assertive communication and teamwork skills with some encouraging results (Lyndon et al., 2012). However, novice nurses are often hesitant to speak up or advocate for patients, particularly in countries such as Japan where there are deep‐seated cultural barriers to assertive communication (Nakamura et al., 2017; Suzuki, Azuma, Maruyama, Saito, & Takayama, 2014). Although training programmes have been introduced to address these issues, there are limited empirical data about the impact of such programmes on novice nurses’ communication behaviours.

This paper presents the evaluation results of an evidence‐based, culturally appropriate, assertiveness communication programme designed to improve nursing students’ level of assertiveness and intention to speak up when concerned about patient safety. For the purpose of this study, assertiveness was defined as “the ability to respectfully express concerns about issues that have the potential to impact patient safety and to share opinions with other staff, including those in authority” (Omura, Maguire, Levett‐Jones, & Stone, 2016).

## 2 BACKGROUND

### 2.1 The need for assertiveness communication training

Assertiveness training was first introduced in the 1950s as a form of behavioural therapy by the American psychologists, Salter and Wolpe (Peneva, & Mavrodiev, 2013). From the 1970s onwards and as a result of the civil rights movement, assertiveness was promoted as a means of protecting individual human rights (Alberti & Emmons, 1974). Since then, assertiveness training has been increasingly used in many fields, including health care.

Traditionally, assertiveness training aimed to improve healthcare professionals’ well‐being, job satisfaction, self‐esteem and workplace relationships (Engin & Cam, 2006; Meng & Sullivan, 2011; Shimizu, Kubota, Mishima, & Nagata, 2004). However, assertive communication training has also been recognized as a critical strategy for addressing escalating concerns about the significant number of errors in health care (Clinical Education & Training Institute, 2011; Thomas et al., 2007).

A lack of assertive communication is a recurring issue in critical incidents. For example, one study investigating the outcomes of root cause analyses of adverse patient incidents in six Danish hospitals identified that healthcare professionals’ hesitance to speak up when concerned about patients accounted for 23% of communication errors (Rabøl et al., 2011). In the United States, communication errors were identified as the root cause of 1796 sentinel events in the years 2013–2015 and a causative factor for delays in treatment, medication errors and incorrect procedures (The Joint Commission, 2016). In Japan, communication failure was identified as a factor in 524 adverse events from 2010–2017 (Japan Council for Quality Health Care, 2017).

Internationally, assertiveness communication training programmes have been introduced to improve healthcare professionals’ communication skills. While the results of a recent systematic review indicated that these types of training programmes are generally effective (Omura, Maguire, Levett‐Jones, & Stone, 2017), there are a limited empirical data about the impact of such programmes on novice nurses’ communication behaviours. In Japan, as in many Asian countries where cultural barriers can hinder assertive communication in health care (Omura Stone, & Levett‐Jones, 2018a, 2018b), this issue is of particular concern.

### 2.2 Theoretical perspective

This study was guided by the Theory of Planned Behaviour (TPB), a conceptual framework frequently used in the design and evaluation of behavioural interventions (Fishbein & Ajzen, 2010). According to the TPB, behaviours are predicted by the strength of the intention to perform a particular behaviour and intentions are influenced by attitude (is it an appropriate thing to do?), subjective norm (is it the right thing to do?) and perceived behavioural control (am I able to do it?) (Ajzen2006a,; Francis et al., 2004).

A meta‐analysis of 185 studies concluded that behavioural intention or motivation is a significant predictor of actual behaviour because people are unlikely to act without motivation (Armitage & Conner, 2001). In health care, the TPB is widely used to predict the behaviours of healthcare professionals and students (Ben Natan, Sharon, Mahajna, & Mahajna, 2017) and as a way of evaluating the potential impact of educational interventions (Lapkin, Levett‐Jones, & Gilligan, 2014; Omura, Levett‐Jones, Stone, Maguire, & Lapkin, 2015). To our knowledge, no previous studies have used the Theory of Planned Behaviour as a theoretical framework to evaluate assertiveness communication interventions. Thus, the current study was designed to evaluate nursing students’:

- behavioural intentions in relation to assertive communication as a result of attending an assertiveness communication training programme;
- attitudes towards speaking up;
- perceptions of the social pressures associated with speaking up for patients (subjective norms); and
- perceived ease of speaking up when concerned about patient safety (perceived behavioural control).

### 2.3 Aim

The aim of this study was to examine the impact of an assertiveness communication training programme on Japanese nursing students’ level of assertiveness and behavioural intention to speak up when concerned about patient safety.

## 3 METHODS

### 3.1 Research design

A quasi‐experimental approach with two parallel groups was used for this study.

### 3.2 Ethical considerations

Approval was obtained from Research Ethics Committees in the researcher's university and the two participating Japanese higher education institutions. Although students attended the workshop as a part of communication subject, completion of the surveys was voluntary and submission of the surveys was taken as implied consent. Participants were assured that their decision to participate or to decline to participate in the study would not disadvantage them in any way. The study is registered in the University Hospital Medical Information Network—Clinical Trial Registry in Japan (UMIN000030276).

### 3.3 Participants

A convenience sample of 150 third‐year Japanese nursing students was recruited for the study. The third‐year students had undertaken several clinical placements and were therefore well positioned to understand and reflect on the importance of assertive communication in health care. A roll of dice was used to allocate one class from each of the two participating institutions into the control group and one into the intervention group. The students in the intervention groups completed surveys after attending the workshop. The second group completed the surveys before attending the workshop and were referred to as the control group. This approach was undertaken to allow comparisons to be made between those students who attended the assertiveness communication workshop and those who had not.

### 3.4 Assertiveness communication workshop

Students in the intervention group participated in a 90‐min assertiveness communication workshop conducted by the researcher. A multi‐method approach was used consisting of pre‐reading, a PowerPoint presentation, videos, group discussion and role‐plays. The workshop was informed by the results of a previous systematic review undertaken to identify the key elements of effective assertive communication training programmes (Omura et al., 2017). It was designed with reference to Gagné’s nine events of instruction (Gagné, Wager, Golas, & Keller, 2005) and Bloom's revised taxonomy (Anderson, & Krathwohl, 2001).

### 3.5 Data collection

Data collection was undertaken from December 2017–January 2018. As well as demographic characteristics, data were collected using the Japanese version of the Rathus Assertive Schedule (J‐RAS) (Suzuki, Kanoya, Katsuki, & Sato, 2007) (with the permission of those authors) and the Theory of Planned Behaviour–Assertive Communication Questionnaire (TPB‐ACQ) which was developed by the authors of the current study.

Rathus (1973) originally developed and validated the RAS self‐report instrument in a study investigating the effectiveness of assertive behaviour training programmes and it has been used in several international studies (Nakhaee, Vagharseyyedin, Afkar, & Mood, 2017; Unal, 2012). The Japanese version of the RAS was validated by Suzuki et al. (2007). The J‐RAS (Suzuki et al., 2007) identifies participants’ level of assertiveness. It has 30 items and scores range from −90 to +90 with higher scores indicating a greater level of perceived assertiveness. The relationship between the J‐RAS and TPB‐ACQ scores was also examined to determine whether there was a relationship between assertiveness levels and speaking up behaviours.

### 3.6 Theory of Planned Behaviour–Assertive Communication Questionnaire

The Theory of Planned Behaviour–Assertive Communication Questionnaire (TPB‐ACQ) was constructed using the steps outlined in Francis et al.’s. (2004) guidelines for the development of TPB questionnaires:

#### 3.6.1 Step I: Belief elicitation study

Individual interviews were conducted to elicit Japanese registered nurses’ (*N* = 23) beliefs and salient behavioural patterns about assertive communication in Japanese healthcare settings (Omura, Stone, Maguire, & Levett‐Jones, 2018). The constructs of the TPB‐ACQ including behavioural beliefs, normative beliefs and control beliefs were informed by the findings of this elicitation study.

#### 3.6.2 Step II: Construction of items TPB‐ACQ

##### Direct behavioural intentions

Behavioural intentions were measured using the intention simulation method recommended by Francis et al. (2004). Three scenarios related to hierarchy, teamwork and knowledge/experience levels were developed to reflect clinical situations where nurses may be required to raise concerns about patient safety. Participants were asked to respond to each intention question with a yes/no answer. The sum of “yes” answers provides the behavioural intention score, with higher scores indicating a stronger intention to speak up.

##### Indirect belief‐based measures

**Figure 1:**
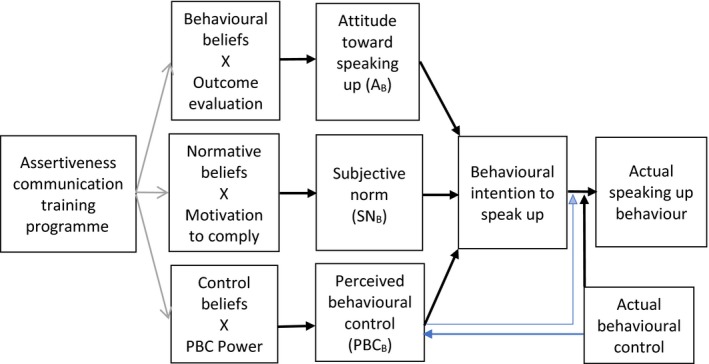
Expected effects of an assertiveness communication training intervention (modified from the Theory of Planned Behaviour diagram (Ajzen, 2006b)—http://people.umass.edu/aizen/tpb.diag.html)

Each scenario was followed by a series of items addressing indirect belief domains of the TPB‐ACQ: attitudes (A_B_), subjective norms (SN_B_) and perceived behavioural control (PBC_B_). Belief‐based attitudes (A_B_) were measured using items that assessed the strength of beliefs about positive or negative consequences of behaviours and whether or not the participant felt that the outcome would be favourable. SN_B_ items assessed the strength of beliefs about the source of social pressure, namely doctors, senior nurses and colleagues and the perceived importance of their approval of the specified behaviour. PBC_B_ items assessed the strength of beliefs about facilitators of and barriers to assertive communication and perceived power to perform the desired behaviour. A_B_, SN_B_ and PBC_B_ scores were calculated by multiplying each belief strength with the corresponding value scale and summing all the multiplied scores (Francis et al., 2004; Figure 1).

#### 3.6.3 Step III: Ensuring cultural appropriateness and face and content validity

The TPB‐ACQ was reviewed for cultural appropriateness by an expert panel consisting of two Japanese academics and five clinicians and items were reworded accordingly. The TPB‐ACQ was also evaluated by six academics and one clinician to ensure face and content validity. Content validity for individual items was 0.86 and over, and the overall content validity for the TPB‐ACQ was 0.98. Thus, no item was removed, but 14 items were reworded to improve clarity.

#### 3.6.4 Step IV: Translation

The first author, who had sociolinguistic/strategic competence (Squires, 2008) in both Japanese and English, translated the questionnaire and the translation was verified by an independent bilingual language specialist. Back translation was then reviewed by a native English‐speaking expert as recommended by Brislin (1970).

### 3.7 Data analysis

Statistical analyses were conducted using SAS v9.4 (SAS Institute, Cary, North Carolina, USA) and SPSS version 24 (IBN corporation, 2018). Data were cleaned and checked in SAS. Data cleaning included assessing the number of missing variables and the data ranges of each variable.

Participant demographics were analysed using the chi‐square test (Table 1). Independent sample *t* tests were conducted to compare differences between control and intervention groups for the following: indirect belief domains of the TPB‐ACQ: attitude (A_B_), subjective norm (SN_B_), PBC_B_, behavioural belief, normative belief, control belief, as well as J‐RAS (Pallant, 2011; Tables 2 and 5). As the behavioural intention score was not a continuous outcome, with values ranging from 0–3, the Wilcoxon Rank sum test was used to compare behavioural intention scores calculated across the three scenarios between the control and intervention groups, rather than an independent *t* test.

**Table 1:** Demographic characteristics

Demographic characteristic (*N* = 123)[Link]	Control (*N* = 65)[Link]	Intervention (*N* = 58)[Link]	*p*‐value from Chi‐square test
Age			
20–21 years	57 (89.1%)	50 (90.9%)	0.739
22 years and older	7 (10.9%)	5 (9.1%)	
Gender			
Male	4 (6.2%)	5 (8.9%)	0.731[Link]
Female	61 (93.8%)	51 (91.1%)	
Prior training			
Yes	1 (1.6%)	1 (1.8%)	1.000[Link]
No	61 (98.4%)	54 (98.2%)	

Frequencies may not add to total sample size due to missing values.

Fisher's exact test used due to small cell sizes.

**Table 2:** Independent sample *t* test comparing belief domains of the TPB‐ACQ between control and intervention groups

TPB domain	Range	Control Mean (*SD*) *N* = 64	Intervention Mean (*SD*) *N* = 58	*t* test for equality of means		
				*t*	*df*	*p*‐value
Attitude (AB)	−84 to +84	10.95 (13.50)	14.19 (12.12)	−1.39	120.00	0.17
Subjective Norm (SNB)	−84 to +84	24.56 (18.09)	18.81 (20.02)	1.67	120.00	0.10
PBCB[Link]	−63 to +63	−11.13 (15.00)	−10.78 (11.00)	−0.15	115.13	0.88
Behavioural belief	1 to 7	4.72 (0.67)	4.82 (0.85)	−0.70	120.00	0.49
Normative belief	−3 to +3	1.09 (0.79)	0.83 (0.86)	1.72	120.00	0.09
Control belief	1 to 7	4.77 (0.84)	4.89 (0.86)	−0.81	120.00	0.42

Pooled method used except Satterthwaite method.

Kendall's tau‐b correlation coefficients and Pearson's product correlation coefficients were used to assess the relationships between TPB‐ACQ belief measures and behavioural intentions and between TPB‐ACQ domains and J‐RAS scores (Tables 4 and 6). P‐values of less than 0.05 were considered to be statistically significant.

## 4 RESULTS

**Figure 2:**
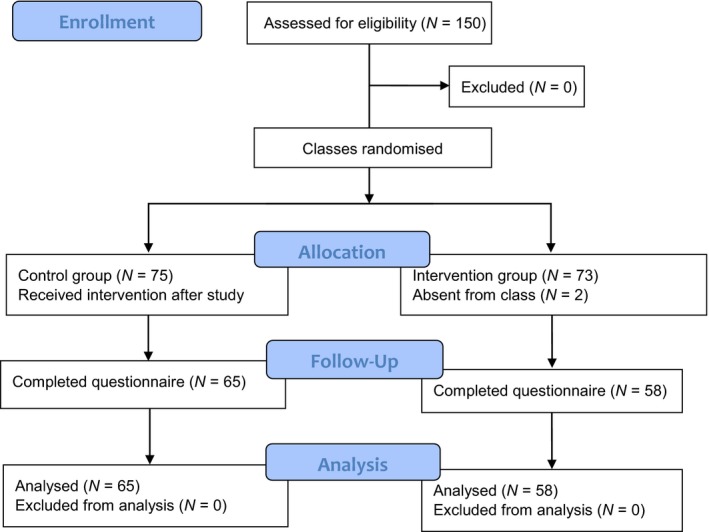
Modified CONSORT 2010 flow chart

We aimed to have a sample size of 65 per group to enable detection of a 0.5 *SD* difference with 80% power and 5% significance. However, of the 150 eligible nursing students, only 123 students participated in this study, resulting in a response rate of 82%. Nevertheless, according to Francis et al. (2004), a sample size of 80 is acceptable assuming at least a moderate effect size (multiple R of 0.3). A flow chart illustrating participant recruitment and participation is presented in Figure 2.

### 4.1 Participant demographics

Study participants included 123 third‐year nursing students aged 20 years or older. Most were female (*N* = 112), recently graduated from high school (*N* = 114) and had no previous assertiveness communication training. There were no significant differences between the demographic characteristics of the intervention and control groups. Participant demographics are presented in Table 1.

Table 2 details the results from the independent sample *t* tests conducted to compare differences in the indirect belief domains of the TPB‐ACQ (A_B_, SN_B_, PBC_B_, behavioural belief, normative belief and control belief) between the intervention and control groups. It describes the mean scores, standard deviation, *t*‐statistic and *p*‐value of both groups for each domain, along with the sample size for each test. The results indicate that there were no significant differences between intervention and control groups for any of the belief‐based domains of the TPB‐ACQ

Table 3 presents the number and percentage of participants from both the control and intervention group who obtained each overall score for behavioural intention (scores ranges from 0–3). For both groups, most participants reported “yes” to all three scenarios; no participant reported “no” to all three scenarios. There was a higher percentage of participants from the intervention group who reported “yes” to all three scenarios compared with the control group (74% vs. 61%). However, the result from the Wilcoxon Rank sum test was not significant (*p*‐value = 0.08) indicating that there was no significant difference between the median behavioural intention scores of the control and intervention groups.

**Table 3:** Frequency and percentage of participants from control and intervention groups obtaining each of the possible values of the overall behavioural intention score

Number of speaking up intentions selected	Control		Intervention	
	%	*N*	%	*N*
0 Yes (3 No)	0.0	0	0.0	0
1 Yes (2 No)	9.4	6	1.7	1
2 Yes (1 No)	29.7	19	24.1	14
3 Yes (0 No)	60.9	39	74.1	43

Table 4 details the results from the correlational analyses assessing the relationship between the indirect belief domains of the TPB‐ACQ (A_B_, SN_B_, PBC_B,_ behavioural belief, normative belief and control belief) and direct behavioural intention score. There was a significant positive relationship between attitudes (A_B_) and intention to communicate assertively. Also, the behavioural belief which corresponds to attitudes (A_B_) had a significant positive relationship with participants’ intention to speak up.

**Table 4:** Kendall's Tau‐b correlation between the belief domains of the TPB‐ACQ and direct behavioural intention

TPB domain	Kendall's tau‐b Correlation coefficient	*p*‐value
Attitude (A_B_)	0.28	0.000
Subjective norm (SN_B_)	0.03	0.653
Perceived behavioural control (PBC_B_)	0.14	0.065
Behavioural beliefs	−0.15	0.047
Normative beliefs	0.05	0.546
Control beliefs	−0.09	0.248

Table 5 shows the mean difference between J‐RAS scores for the intervention and control groups. Participants in the intervention group had higher mean assertiveness scores than the control group; however, this difference was not significant.

**Table 5:** Independent *t* test comparing mean J‐RAS scores for control and intervention groups

Variable	Range	Control Mean (*SD*) *N* = 64	Intervention Mean (*SD*) *N* = 58	*t* test for equality of means		
				*t*	*df*	*p*‐value
Assertiveness level	−90 to +90	−17.59 (23.13)	−10.70 (21.11)	−1.71	120.00	0.09

Table 6 details the results from the correlational analyses assessing the relationship between the domains of the TPB‐ACQ (behavioural intention, A_B_, SN_B_, PBC_B,_ behavioural belief, normative belief and control belief) and J‐RAS score. Direct behavioural intention and PBC_B_ had a significant relationship with the participants’ level of assertiveness.

**Table 6:** Correlation between TPB‐ACQ and J‐RAS scores

TPB domain	Correlation coefficient	*p*‐value
Behavioural intention[Link]	0.18	0.014
Attitude (A_B_)[Link]	0.14	0.111
Subjective norm (SN_B_)[Link]	−0.16	0.088
Perceived behavioural control (PBC_B_)[Link]	0.19	0.033
Behavioural beliefs[Link]	−0.02	0.791
Normative beliefs[Link]	−0.15	0.097
Control beliefs[Link]	−0.10	0.293

Kendall's Tau‐b correlation was used to assess relationship between J‐RAS total score and behavioural intention.

Pearson product correlation was used to assess relationship between J‐RAS total score and belief domains of TPB‐ACQ.

## 5 DISCUSSION

This study sought to evaluate the impact of an assertiveness communication training programme on Japanese nursing students’ behavioural intentions and level of assertiveness. We anticipated that the training programme would promote the development of a personal conviction regarding the importance of assertive communication and, as a result, the intention to speak up in a clinical setting if confronted by an issue that may influence patient safety. The effectiveness of the assertiveness communication training programme was evaluated using the TPB‐ACQ, an instrument purposively designed for this study and the Japanese version of the Rathus Assertiveness Schedule (J‐RAS; Suzuki et al., 2007).

Many previous studies have demonstrated that the most important determinant of behaviour is intentions and that behavioural intentions can be used as a proxy for actual behaviours (Montano & Kasprzyk, 2015). It was hoped that participants would be highly motivated to speak up when needed as a result of their attendance at the assertiveness communication workshop that focused specifically on patient safety. The results of the TPB‐ACQ did identify that there was a higher percentage of students from the intervention group who indicated that they would speak up when concerned about patient safety than in the control group; however, this result was not significant.

In the scenario about speaking up about a doctor's poor hand hygiene which addressed issues of hierarchy, professional status and gender imbalance, a large proportion of participants from both groups indicated that they would not speak up. It is likely that participants were strongly influenced by Japanese cultural norms and the perception that, within the traditional hierarchy of healthcare environments, doctors remain unapproachable. In addition, it is possible that the consequences of poor hand hygiene are not immediately evident, an issue which often makes it difficult to change hand hygiene behaviours (WHO, 2009). These results suggest that in future assertiveness training programmes, greater attention to culture and hierarchy is needed, along with a strong show of support from medical staff, to overcome the challenges associated with differences in professional status.

In a previous study informed by the TPB that evaluated the impact of an interprofessional multimedia learning programme, the results indicated that Japanese registered nurses had higher intention scores than nursing students (Omura et al., 2015). This result may have been influenced by the fact that in Japan, nursing students have minimal clinical exposure, most of which is observational in nature. There are therefore few opportunities to work with or learn from other members of the healthcare team. Consequently, in the current study, it is possible that the participants may not have contemplated standing up to doctors or other senior staff and that this was not something that they could envisage doing in the future.

Understanding the relationship between each of the TPB‐ACQ variables and students’ intention to speak up is important for the improvement of future educational interventions. In this study, although participants in the intervention group had higher A_B_ and PBC_B_ scores, the difference was not significant. Conversely, the intervention group had lower SN scores. While the reasons for these results are difficult to determine, it should be noted that there was a significant positive relationship between attitudes (A_B_) and intention to speak up. This suggests that students’ attitudes may be a determining factor in their intention to speak up when concerned about patient safety. As attitude is generally found to be the strongest predictor of intention in TPB‐based studies (Armitage & Conner, 2001), this result is encouraging and attests to the importance of continuing to focus on attitudinal change in future assertiveness communication training programmes (Montano & Kasprzyk, 2015).

It should be noted that mean scores for the behavioural belief item (range 1–7) about assertive communication having a positive impact on patient safety were high for both the control and intervention groups (control mean = 6.17, *SD* = 1.06; intervention *M* = 6.19, *SD* = 1.07) leaving little room for overall improvement (Ajzen, 2011). Similarly, mean scores for the behavioural belief item about disadvantages of speaking up behaviour and causing group disharmony were also relatively high for both groups (control *M* = 4.40, *SD* = 1.55; intervention *M* = 4.50, *SD* = 1.74). This might have been due to the strong influence of Japanese cultural value of collectivism (Omura Stone, & Levett‐Jones2018a,; Omura et al., 2018).

Direct behavioural intention and belief‐based perceived behavioural control (PBC_B_) were found to have a significant positive relationship with students’ level of assertiveness. PBC affects not only motivation but also behaviour since action is influenced by external factors such as time and the person being spoken to (Ajzen, 1991). Therefore, it is noteworthy that nursing students who attended the workshop demonstrated stronger PBC_B_ and corresponding control beliefs than those who did not attend. However, as shown in Table 2, the mean for PBC_B_ was in the negative range for both control and intervention groups and the lowest of the indirect composite constructs. This is due to low PBC value scales (PBC power), or in other words, how barriers make it more difficult for students to speak up. Among those barriers, nursing students’ lack of knowledge and experience were perceived most negatively, followed by the “busyness” of the target person that made it difficult for the student to speak up. Therefore, despite the belief that assertive communication improves patient safety, as shown by positive mean for belief‐based attitude (A_B_), students may not have thought that it was achievable because they lacked the skills to be assertive. This result reinforces the importance of ongoing training and practical opportunities to learn and be assessed on assertive communication skills.

The intervention group displayed a lower belief‐based subjective norm (SN_B_) and a corresponding normative belief compared with the control group. SN_B_ or perceived social pressure had the weakest positive relationship with overall behavioural intentions among three composite variables in this study. Although this relationship was not significant, this is consistent with the evidence from a meta‐analysis that identified that SN is the weakest predictor of intentions among TPB constructs (Armitage & Conner, 2001). However, it should be noted that the mean scores for SN_B_ were high in both the intervention and the control group, possibly due to the strong motivation to comply with important referents such as doctors and senior nurses. The Japanese emphasis on harmony and conformity may also have contributed to this result (Omura Stone et al.2018a,; Omura et al., 2018). Unlike personal factors such as attitudes or internal factors of PBC such as confidence, SN cannot easily be changed. The workshop sought to address this issue by including messages of support from nursing leaders, a strategy which is considered vital to changing organizational culture (Okuyama, Wagner, & Bijnen, 2014; Sayre, McNeese‐Smith, Leach, & Phillips, 2012). However, follow‐up postregistration training would be helpful to further modify SN because of the significant influence of organizational cultures.

The J‐RAS results indicated that participants in the intervention group who attended the assertiveness communication workshop had higher mean assertiveness scores than those in the control group. However, this result was not significant. This is somewhat disappointing; however, it is noteworthy that the overall assertiveness level of the participants was in the negative range and much lower than those of students from other countries, including those from non‐Western countries such as India and Turkey (Arslan, Akça, & Baser, 2013; Nirmala & Suni, 2016). These results reinforce the need for ongoing assertiveness training in Japanese undergraduate nursing programmes, as well as further research to determine the effectiveness of such interventions (Deltsidou, 2009; Timmins & McCabe, 2005).

### 5.1 Limitations

The results of this study may have been influenced by several limitations. For example, social desirability bias in regard to the Japanese cultural norm of wanting to live up to social expectations (De Mente, 2004) may have influenced the results. Although background factors such as personality are not expected to influence TPB results (Montano & Kasprzyk, 2015), adjusting groups by taking these factors into consideration at the baseline may have led to more accurate evaluation of the intervention. Additionally, no pre–post intervention comparison, which might have shown an immediate effect of the workshop, or follow‐up data collection was made in this study.

As the Cronbach's alpha was below 0.6 for the three direct TPB domains, composite variables of the direct TPB domains were not analysed. Instead, the means for each item of the direct TPB constructs between the groups were compared. The Wilcoxon Rank sum test was used to compare control and intervention participant's median behavioural intention instead of independent *t* tests due to the ordinal nature of this outcome. Lastly, actual participant behaviours were not measured. Therefore, transfer of behavioural intention to actual assertive behaviour in practice cannot be determined.

### 5.2 Implications for further research

The assertiveness communication training programme used in this study could be modified for use in other countries with similar cultural contexts or with a culturally diverse nursing workforce. However, the impact of longer or repeated training sessions should be evaluated, along with the impact of an integrated and/or scaffolded approach to assertiveness communication training in undergraduate nursing programmes. Additionally, future programmes should continue to focus on attitudinal shifts as a motivator for behaviour change, with particular attention to addressing issues associated with patient safety. Lastly, studies should focus on strengthening students’ beliefs about their capacity to communicate assertively and examining the impact of culture on communication.

## 6 CONCLUSION

Despite an increasing focus on healthcare professionals’ teamwork and assertive communication skills as a strategy to improve patient safety, evidence suggests that cultural barriers exert a significant influence on communication behaviours, especially for novice nurses. This study sought to design, implement and evaluate the impact of a culturally appropriate, evidence‐based assertive communication training programme for nursing students. The results demonstrated that the programme had a positive impact on levels of assertiveness, perceived behavioural control and attitudes towards assertive communication and suggested that these types of interventions have the potential to improve nursing students’ assertive communication skills and ultimately patient safety. However, future studies should focus on patient safety as a motivator of behaviour change, as well as the impact of ongoing training and evaluation of transfer to practice.

## CONFLICT OF INTEREST

We declare no conflict of interest.

## AUTHOR CONTRIBUTION

All authors have agreed on the final version and meet at least one of the following criteria: Substantial contributions to conception and design, acquisition of data, or analysis and interpretation of data; Manuscript writing and revisions for important intellectual content.
